# How should we implement collaborative care for older people with depression? A qualitative study using normalisation process theory within the CASPER plus trial

**DOI:** 10.1186/s12875-018-0813-7

**Published:** 2018-07-18

**Authors:** Anna Kathryn Taylor, Simon Gilbody, Katharine Bosanquet, Karen Overend, Della Bailey, Deborah Foster, Helen Lewis, Carolyn Anne Chew-Graham

**Affiliations:** 1 Faculty of Health Sciences, University of Bristol, Senate House, Tyndall Avenue, Bristol, BS8 1TH UK; 2 Mental Health and Addictions Research Group (MHARG), Department of Health Sciences, University of York, York, YO10 5DD UK; 3 Research Institute, Primary Care and Health Sciences, Keele University, Keele, Staffordshire ST5 5BG UK; 4 West Midlands CLAHRC (Collaboration for Leadership in Applied Health Research and Care), Warwick, UK

**Keywords:** Older people, Depression, Collaborative care, Qualitative analysis, Normalization process theory

## Abstract

**Background:**

Depression in older people may have a prevalence as high as 20%, and is associated with physical co-morbidities, loss, and loneliness. It is associated with poorer health outcomes and reduced quality of life, and is under-diagnosed and under-treated. Older people may find it difficult to speak to their GPs about low mood, and GPs may avoid identifying depression due to limited consultation time and referral options for older patients.

**Methods:**

A qualitative study nested within a randomised controlled trial for older people with moderate to severe depression: the CASPER plus Trial (Care for Screen Positive Elders). We interviewed patient participants, GPs, and case managers (CM) to explore patients’ and professionals’ views on collaborative care developed for older people, and how this model could be implemented at scale. Transcripts were analysed thematically using normalization process theory.

**Results:**

Thirty-three interviews were conducted. Across the three data-sets, four main themes were identified based on the main principles of the Normalization Process Theory: understanding of collaborative care, interaction between patients and professionals, liaison between GPs and case managers, and the potential for implementation.

**Conclusions:**

A telephone-delivered intervention, incorporating behavioural activation, is acceptable to older people with depression, and is deliverable by case managers. The collaborative care framework makes sense to case managers and has the potential to optimize patient outcomes, but implementation requires integration in day to day general practice. Increasing GPs’ understanding of collaborative care might improve liaison and collaboration with case managers, and facilitate the intervention through better support of patients. The CASPER plus model, delivering therapy to older adults with depression by telephone, offers the potential for implementation in a resource-poor health service.

## Background

The prevalence of depression in older people is estimated to be as high as 20% [1, 2]. This high prevalence may be due to increased prevalence of long-term physical conditions in this age group: depression is often co-morbid with long-term conditions such as ischaemic heart disease, diabetes, stroke and Parkinson’s disease [3], and leads to poorer health outcomes [4]. Depression causes a significant functional impairment, with reduced quality of life and increased risk of suicide [5]. This results in a socioeconomic burden, compounded by increased use of health and social care, including unscheduled care [6].

Identifying and managing depression in older people is often challenging [7–9]. Older people with chronic physical illness often normalize their depression, or view it as a justifiable cause of low mood [7, 10, 11]. They may be reluctant to define low mood as a mental illness because of the perceived stigma associated with a diagnosis of depression. As a result, older people may hold negative views about help-seeking [8]. Management can focus on the biomedical model, with less emphasis on patients’ social, cultural or economic background which could have informed management [7]. For example, participation in meaningful activities has an important role in improving quality of life [12] and mental health and wellbeing in later life [13–15]. For older people with undetected depression, longer-term prognosis is poorer than those whose depression is known by their GP [16].

Older people are a vulnerable and under-served group, often experiencing difficulty in accessing mental health care [17]. This is compounded by social isolation, loneliness, and economic deprivation [18, 19]. Given the aging population and the public health implications of depression in older people, it is clear that acceptable community interventions focused on older people with depression are needed [20].

Behavioural activation is an effective brief psychological intervention for people with depression [21–23]. Several trials have evaluated the effectiveness of behavioural activation within a collaborative care model, with psychological wellbeing practitioners supporting patients [24–26]. The collaborative care model incorporates a multi-professional approach to patient care with enhanced communication between professions, a structured management plan and scheduled patient follow-up encounters [27]. Case managers may be able to reduce the stigma of a diagnosis of a mental illness and resolve misconceptions around antidepressant medication prescribed by GPs [28].

There is a good evidence-base to support the use of collaborative care in managing people with depression [29]. Thus, the UK CADET study reported that, in a general adult population, the positive effects of collaborative care were maintained up to 12 months after initiation of the intervention [24, 30], and the UK COINCIDE study of collaborative care for adults with diabetes or cardiovascular disease and co-morbid depression also reported positive results [25]. For older adults, an American study demonstrated the effectiveness of collaborative care [31], and the UK CASPER plus trial [32] evaluated the clinical- and cost-effectiveness of collaborative care for older people with moderate to severe depression [24]. The first qualitative study nested within the CASPER plus trial reported that being invited to participate in a trial about depression seemed to allow older people to disclose their feelings, name the problem, and seek help. Offering older people an opportunity to talk outside the primary care consultation was valued by patients and GPs, and behavioural activation delivered by a case manager in the primary care setting filled a gap in the care of older people with depression [11]. However, how a collaborative care intervention for older people with depression can be implemented on a wider scale has not yet been reported.

We report further analysis of data generated in the nested qualitative study within the CASPER plus trial [26], which explores patients’ and professionals’ views on collaborative care and how this model could be implemented at scale.

## Methods

We conducted analysis of the qualitative data generated using semi-structured interviews nested within the CASPER plus (Collaborative Care for Screen Positive Elders) pragmatic randomized controlled trial [24], using normalization process theory as a framework for analysis [32, 33].

The CASPER plus RCT recruited 584 participants aged 65 years and older with major depressive disorder. Exclusion criteria were known alcohol dependency, known symptoms of psychosis, known co-morbidity making entry to the trial inadvisable (such as recent self-harm or significant cognitive impairment), or other factors making trial entry inappropriate such as recent bereavement or terminal malignancy. The intervention arm received a low-intensity intervention of collaborative care delivered by a case manager for an average of six sessions over 7–8 weeks alongside usual GP care, while the control arm received usual GP care. The collaborative care intervention included five components: patient centred assessment (in which the patient was assessed in their residence by the case manager, focusing on the presence and severity of depressive symptoms, and information given), symptom monitoring (across all subsequent patient contacts), medication management (anti-depressant prescribing at the discretion of the GP but the case manager encouraged concordance and addressed patient concerns), active follow-up (by the case manager, either face to face or on the telephone), and behavioural activation (offered by the case manager, following a structured programme). The primary outcome was self-reported symptoms of depression, assessed by the Patient Health Questionnaire-9 (PHQ-9) at four months post-randomisation, and also at 12 and 18 months.

### Ethical approval

Leeds East Research Ethics Committee, Yorkshire & Humber, gave ethical approval for the RCT and this qualitative study (reference 10/H1306/61).

### Recruitment and sampling

We aimed to interview participants from three groups: GPs within CASPER plus trial practices, case managers delivering the intervention, and patients (including both participants who completed, and those who withdrew from the intervention). All case managers were invited to be interviewed once they had delivered a course of treatment to at least three participants, and GPs from practices with at least five participants from the collaborative care arm of the trial were invited to be interviewed. Once we had recruited approximately half of our participants this way, we then used a purposive sampling strategy with the aim of gaining a more varied sample of patient and GP participants. This sample included participants from both urban and rural areas in the North of England, with the aim of generating data from patients and GPs in areas of differing levels of deprivation. In addition, we ensured a spread in age, gender and socioeconomic status. We invited all participants who dropped out of the intervention to participate in an interview.

Invitation letters, a participant information leaflet describing the interview process, and a consent form with a stamped envelope were sent to patient trial participants by post. GPs and case managers were sent an invitation letter, consent form and participant information leaflet by email. Written informed consent was obtained from all participants prior to interviewing.

GP practices recruiting participants invited eligible patients aged 65 years and over to participate in the CASPER plus trial [32]. Once five or more patients from each practice had completed the intervention, the lead GP was invited to be interviewed in the qualitative study.

All patient participants were invited to be interviewed after they had completed the intervention. Non-responders in both groups were followed up by telephone. We attempted to recruit two groups of participants: those who completed the intervention and those who withdrew.

At the start of our study, all invited participants were from urban and rural practices in Harrogate, York, Hull and surrounding areas due to the sequence of GP practice recruitment. These are areas of relatively low to moderate deprivation, so we then used purposive sampling to ensure that participants from areas of higher deprivation were invited to be interviewed.

### Data collection

Interviews were conducted by KB, KO and SN at a time and location convenient to the participant. For GPs, this was at their practice; for participants, interviews were carried out at their home; for case managers, interviews were done at the researcher’s office. Interviews were completed between May 2013 and November 2014. With participants’ consent, all interviews were digitally recorded, transcribed verbatim, and anonymised. The topic guides were developed with reference to the existing literature and to the principles of normalization process theory (Table 1). They were discussed and agreed within the research team; the guides were modified as data generation and analysis proceeded.

**Table 1:** The four key elements of Normalisation Process Theory (from May 2009 [33] and www.normalizationprocess.org)

*Coherence*: a set of ideas about the meaning, uses and utility of a practice, (defined as an ensemble of beliefs, behaviours, and acts that manipulate or organize objects and others), which hold the practice together and make it possible to share and enact it. This is the *sense-making* work that people do individually and collectively when they are faced with the problem of operationalizing some set of practices	
*Cognitive participation*: the symbolic and real enrolments and engagements of human actors that position them for the interactional and material work of collective action. This is the *relational* work that people do to build and sustain a community of practice around a new technology or complex intervention	
*Collective action*: the chains of interactions which are the site of mental and material work to organise and enact practice which might include reshaping behaviours or actions, employing objects or artefacts, or reorganising relationships and contexts.This is the *operational* work that people do to enact a set of practices, whether these represent a new technology or complex healthcare intervention	
*Reflexive monitoring*: the continuous evaluation, both formally and informally, of implementation processes by participants, which may involve judgments about the utility and effectiveness of a new practice with reference to socially patterned and institutionally shared beliefs This is the *appraisal* work that people do to assess and understand the ways that a new set of practices affect them and others around them	

### Data analysis

An initial thematic analysis has been reported elsewhere [11]. Following this, a theory-driven framework analysis [34] was conducted, led by AKT and CCG who independently analysed the transcripts, utilizing the normalization process theory (NPT) [33], analysis being guided by the four main constructs of NPT (coherence, cognitive participation, collective action and reflexive monitoring, see Table 1). This enabled the identification of similarities and differences between transcripts, and the noting of negative cases, before focusing on relationships between the data. Data from the different perspectives (GPs, CMs, patients) were considered individually and then compared with data from other recruits from the same perspective (i.e. GP interviews were compared with GP interviews), and then the group frameworks were compared to each other. Following initial analysis the framework was agreed across the wider research team, including researchers of different professional backgrounds (including health sciences research and academic primary care) to enhance rigor [35].

## Results

Of the 18 GPs invited to take part, 12 consented to be interviewed. Eight of the 12 case managers who took part in the trial agreed to be interviewed, all of whom had delivered the intervention to at least three patient participants. Twelve patient participants who had completed the intervention (out of 18 invited) agreed to be interviewed, one person who had withdrawn from the intervention prior to starting therapy agreed to be interviewed. The following tables give the demographic characteristics of the interview participants (Tables 2, 3 and 4).

**Table 2:** Demographics of case managers

Gender	Years of experience^a^	Interview type (face to face or telephone)
F	8	Face to face
F	9	Face to face
F	4	Face to face
F	4	Face to face
F	4	Telephone
F	3	Telephone
F	3	Telephone
F	5	Face to face

^a^Experience in years of delivering a low-intensity psychological intervention

**Table 3:** Demographics of GPs

Gender	Practice size	IMD^b^	Rural or urban GP practice
M	14,886	5	Urban
M	10,150	6	Urban
M	19,879	10	Rural
F	18,083	8	Rural
M	24,353	5	Urban
M	15,915	4	Urban
M	6961	6	Urban
F	13,000	3	Urban
F	18,083	8	Rural
F	11,893	6	Rural
M	7183	10	Rural
M	15,432	5	Rural

^b^Index of Multiple Deprivation. Lower numbers indicate lower socioeconomic status

**Table 4:** Demographics of patient participants

Gender	Age range	IMD^b^	Interview type (Face to face or telephone)	From rural or urban GP practice
F	75–80	1	Face to face	Urban
M	75–80	9	Face to face	Urban
M	65–70	5	Face to face	Rural
M	81–85	8	Face to face	Rural
M	65–70	2	Face to face	Urban
F	65–70	10	Face to face	Rural
F	65–70	10	Face to face	Rural
F	65–70	10	Face to face	Urban
M	65–70	2	Face to face	Urban
F	65–70	8	Telephone	Urban
F	75–80	9	Face to face	Urban
F	65–70	9	Telephone	Urban
M	65–70	6	Face to face	Rural

^b^Index of Multiple Deprivation. Lower numbers indicate lower socioeconomic status

Eight case managers out of the 12 trained interviewed; the four case managers who declined all worked at the site which was last to join the study; and had been allocated fewer trial patients than other case managers. Thirteen patient participants (of whom 12 had completed the intervention and one withdrew before starting therapy) were interviewed. It was challenging to recruit older people who had withdrawn from the trial, as most had done so at the outset by either declining to receive the intervention or withdrawing after a single session. They then declined to participate in an interview to discuss their decision.

The main themes that will be presented in this manuscript are: understanding of collaborative care, interaction between patients and professionals, liaison between GPs and case managers, and the potential for implementation of the CASPER plus intervention in UK primary care.

Data is presented to support analysis, labelled by identifier and number where: CM = case manager; PT = patient; GP = general practitioner.

### Understanding of collaborative care (coherence)

Case managers (CMs) described a working understanding of the CASPER plus trial and the collaborative care model.

*‘Collaborative care with the participant, and collaborative care with the GP, so you work in kind of a triad.*’ [CM4]

Most CMs understood the structure and content, and potential value, of the intervention, and their role in delivering it:

*‘With collaborative care that the, the person, either the patient or the participant is more central to that and there’s more, there’s more kind of two-way communication, I suppose, whereas if you just normally standard care you would have the GP liaising with somebody and then that would, that person would get in contact with the patient.’* [CM2]

Many GPs reported that they understood what the CASPER plus trial, in which their practice was participating, involved, although were not clear on the detail of the intervention being delivered:

*‘I understand a number of visits via, I think it’s eight visits, via a trained case manager over a period of time.’* [GP7]

*‘I must admit, not actually experiencing it I didn’t really quite know what was involved with it.’* [GP5]

GPs were keen to highlight their views on the potential benefit of the case manager intervention:

*‘I would see it as yes we sort of complement each other really and what it does it sort of positively reinforces what we do but also picks up on stuff perhaps that we may have missed because of what I‘d mentioned with regards to constraints within general practice at the moment.’* [GP10]

Few patients talked about their understanding of the collaborative care model, rather focusing, perhaps not unsurprisingly, on their interactions with the case manager:

*‘Some of it was to me useful… but the basic concept of it I am not at all sure about. I still don’t know what collaborative care is… Still, err, if usual care means you get something out of your GP when you go along, collaborative care means someone takes some sort of initiative and checks up on you from time to time. But who is doing the collaboration, obviously I am doing part of, I am one part of the collaboration but who is the other part?’* [PT4]

*‘Having someone to talk to… about things in my life that I would talk to say the family about or friends unless they were extremely close friends, it gave me someone objective to talk to you know, that was removed from my situation.* [PT2]

### Interaction between patients and professionals (cognitive participation)

Both patients and case managers felt that the initial face-to-face meeting was essential, but could be followed successfully by telephone interactions.

*‘I think doing the work over the phone maybe if you’ve done, you know the first appointment face to face you’ve maybe got an understanding of some of those areas and then a lot of the stuff you can still deliver over the phone.’* [CM7]

*‘Well it is nice to know who you are talking to to start with, not just a voice at the other end but once you know that voice I can put a face to it and then… I think it was better on the telephone to be quite honest, concentrating more.’* [PT3]

However, communication difficulties were highlighted as potential barriers in this patient group.

*‘I was in a really bad way, weeping and feeling lousy and not being able to do much at all. I probably think in a case like that it may – to some it may work okay on the phone, but I think for probably a person like me, face-to-face contact is more helpful.’* [PT9]

*‘I have to say the telephone conversations were very difficult, because the line was very bad. I don’t know whether she used a mobile or whether it was from an office or an extension, but it was very, very difficult to hear her most of the time. So it was a bit of a strain in that. But if I’d been able to hear her better, the telephone worked just as well as the face-to-face. [But you need face-to-face first because] you need to identify who you’re talking to, and that helps to focus on who you’re speaking to, really.’* [PT7]

Patient participants suggested that it was valuable for their case manager and GP to liaise, and for their GP to receive patient progression summaries, which they had experienced of being acted upon.

*‘If it isn’t urgent I should say just to write, but they’re very, very good that if you ring up and speak to the receptionists the doctors ring you back.’* [PT1]

### Liaison between case managers and GPs (collective action)

GPs and CMs recognized that they needed to liaise with each other, both as part of the Collaborative Care framework, but also to improve patient care, and welcomed the other professional’s input.

*‘I think it’s really important that the case manager has a relationship with the GP as well as the participant so I think it’s really important whenever anything like this is set up in a practice that the case manager is part of it in that setup process. I’m not saying that the case manager has to go and see the GP every week or anything like that but they have to know who they’re talking to.’* [CM1]

*‘I would certainly look forward to seeing letters coming back after a few sessions that give some feel as to what sort of progress is being made and then again at completion I think.’* [GP7]

As patients had also expressed, this liaison was felt to be particularly important when communicating patient risk.

*‘One gentleman that I saw, he said that the most useful thing had been that diagnostics and risk was identified and so we wrote to the GP about that. And it was the risk was still there when I saw him for the first time so I put that in a letter as well and he said that that had kind of opened the door. He would have never gone and spoken to his GP about it but he felt that now the GP had been informed that he was happy.’* [CM2]

However, both GPs and case managers reported difficulties in being able to communicate reliably with each other, due to CM perceptions about GPs’ working hours and the volume of letters and phone calls they already receive, along with GPs’ concerns about increasing workload.

*‘So when I have had contact with the GPs… if they’ve not been there when I call, then it has been quite difficult, and we tend to keep missing each other, that kind of thing.’* [CM6]

*‘If someone was to ring me say at three o’clock and say well can you ring me back before five that’s going to be pretty impossible because I’m just you know I’ve just got one patient after another but I could ring them back you know the following morning or that type of thing so that would work. Or email.’* [GP1]

*‘I would say the only thing with letters is that they’ll often sit for a while, while we get through them all really.’* [GP2]

### Evaluating collaborative care (reflexive monitoring)

Participants suggested a variety of barriers and facilitators to delivering the intervention to larger groups of people. Case managers felt that it was important for GPs to have a greater understanding of collaborative care.

*‘Maybe a bit more education for GPs and a bit more networking and stuff like that, telling each other what they’re doing and stuff like that could be more helpful.’* [CM3]

GPs suggested that CMs should be attached to, or embedded in, practices to improve liaison and communication.

*‘I know if somebody came to our practice and said, “I’m the case manager to do this, and these are the sort of people that I want to see,” we’d love it. If that was provided, I think that would be a really, really good service. And as I said, the case managers that we’ve had, when we remember that they’re there, they’re brilliant. It’s really nice when you keep going to see the same person with the same kind of things to just think, “Well, if I can get that person in, they can go and see them, have a really long period of time with them, and actually get a handle on things and sort things out.” I think we would just love to do that.’* [GP8]

Similarly, CMs felt that being able to review patients with GPs would enable better care, although they recognized that this added an additional time commitment to both the case manager and the GP.

*‘I think [a joint review would] be a good idea but it’s just time isn’t it and like when you’re lumped with, because I’ve worked in practice before when you’ve got like massive caseloads of people and then you’ve got like this extra, it sounds really horrible but when you’ve got this extra, you know like, review to do as well and then that needs, you know it’s just… I think that would be good for [the patient] because again it’s all about liaising and people know about what’s going on with them and make them feel more cared for, I think you know it’d be good for them.’* [CM4]

## Discussion

### Summary of findings

This qualitative study, utilizing the principles of the Normalisation Process Theory [33] explores patients’ and professionals’ views on collaborative care for older people with depression, and suggests how this model could be implemented at scale.

We found that, the case managers delivering the intervention as part of a randomised controlled trial regarded collaborative care as a coherent model to work with. The GP respondents, however, had a more vague understanding of what the collaborative care model entailed, and what constituted the patient-level intervention. Patient participants’ accounts focused on the one-on-one interaction with the case managers rather than the intervention itself, although they did report the communication between their CM and GP as a positive.

The collective action required to implement collaborative care within a general practice was made difficult by GPs’ lack of understanding of the collaborative care framework. Although professionals reflected positively on the potential benefits of implementing a collaborative care approach (reflexive monitoring), GPs reported that they did not fully understand the CC model and that they had little communication with the case manager. This suggests that this model of care did not impact on their routine work, and we did not achieve collaboration as much as we had hoped within the context of the trial. Case managers suggested that an opportunity for joint consultations, with the GP and patient present, might improve liaison between CM and GP, and ensure that the behavioural activation intervention could be reinforced.

Older people with depression within the CASPER plus study valued the initial face-to-face session with the case manager, and, the majority suggested that further contact with the CM by telephone was acceptable. People with hearing problems, however, commented on the difficulties using the telephone, expressing a preference for continuing face-to-face sessions with the CM.

### Comparisons with previous literature

This study was nested within the CASPER plus trial, which showed that a collaborative care framework in which to deliver a behavioural activation intervention to older people with depression is effective in the short term, though the reduction in depression severity was not maintained over the longer term of 12 or 18 months. However, participants who received six or more sessions of collaborative care did benefit substantially more than those who received fewer treatment sessions [32]. The NPT analysis builds on the initial thematic analysis of the data reported by Overend et al. [11], suggesting that invitation to participation in a trial revealed hidden depression and identified blind spots previously hidden from both GPs and patients. Liaison between health care professionals is a key feature of a collaborative care framework, and, as with previous literature [36], opportunities for liaison and collaboration between CMs and GPs were reported to be limited by the respondents in this study. CMs suggested that increasing GPs’ understanding of the nature of the intervention being delivered in the CASPER plus trial [26] might increase opportunities for collaboration, and that if this model of care were implemented widely, such liaison would be necessary in order to ensure education for GPs about collaborative care. GP respondents in this study were positive about the prospect of working more closely with case managers, suggesting that locating them within practices would be important in order to foster greater collaboration. This is supported by previous work [37].

The CMs interviewed suggested that joint reviews with the GP and patient would be a way of increasing communication and liaison. A similar model was integral to the COINCIDE trial [25] with case managers and practice nurses holding joint consultations with patients with diabetes or heart disease and depression [38]. Key findings of this study suggest that care was felt to be better co-ordinated but patients still preferred their mental and physical problems to be treated separately. This is important for older people where mental and physical co-morbidity is common.

### Strengths and limitations

A key strength of this study includes the use of qualitative methodology to enable us to explore, in detail, multiple perspectives, views and experiences of participants within the CASPER plus trial [26], offering an opportunity to explain the trial results. This helps to facilitate interpretation and implementation of trial findings [39]. Our research therefore adds to an emerging literature using embedded qualitative methods in RCTs to understand the role and value of collaborative care.

Further analysis of data using the four constructs of NPT (Coherence, Cognitive Participation and Reflexive Monitoring) adds to the value of the data generated, and provides important information on the range of participants (patients and professionals) within the trial and what might be the barriers and facilitators to implementation of the collaborative care model in routine primary care. Analysis was conducted by a multi-professional team (primary care, psychology, psychiatry), which contributes to trustworthiness of analysis [35].

Limitations of the study include the range of participants interviewed: We had aimed to interview people across a wide demographic range, but were less successful at recruiting those from areas of lower socio-economic status, which may be due to levels of social deprivation and difficult financial circumstances restricting participants from entering the trial and then the interview. Similarly, GPs from areas of lower socioeconomic status were less likely to respond to an invitation to be interviewed. We therefore may have missed additional barriers and facilitators to implementation that are specific to populations of lower socioeconomic status. The case managers who joined the trial last, and worked in more deprived areas, did not agree to be interviewed, and we also had difficulty recruiting participants who withdrew from the intervention. We could have missed other facilitators or barriers to implementation due to this.

The semi-structured topic guide offered flexibility with questioning and the ability to modify prompts as the study progressed. However, it is possible that some prompts may have limited participants to a specific answer, and therefore biased our findings.

### Implications

A telephone-delivered intervention to older people with depression would be acceptable to older people, following an initial face-to-face meeting, and offers the potential for implementation in a resource-poor health service. The need for liaison between CMs and GPs is emphasized both as an integral part of the CC model, but also recognized by.

Implementation would require improved buy-in from GPs. Facilitators of this could include co-location of CMs within practices [36, 37] utilizing technologies such as telephone, skype and e-mail.

## Conclusions

A telephone-delivered intervention, incorporating behavioural activation, is acceptable to older people with depression, and is deliverable by case managers. The collaborative care framework makes sense to has the potential to optimize patient outcomes, but implementation requires integration in day to day general practice. Increasing GPs’ understanding of collaborative care might improve liaison and collaboration with case managers, and facilitate the intervention through better support of patients. The CASPER plus model, delivering therapy to older adults with depression by telephone offers the potential for implementation in a resource-poor health service.

## Abbreviations

CASPER plus: Collaborative Care for Screen Positive Elders
CM: case managers
GP: general practitioner
NPT: normalization process theory
